# Nurse led home-based care for people with HIV/AIDS

**DOI:** 10.1186/s12913-018-3002-4

**Published:** 2018-03-27

**Authors:** Elizabeth M. Wood, Babalwa Zani, Tonya M. Esterhuizen, Taryn Young

**Affiliations:** 1 Clinical Sciences, Liverpool School of Tropical Medicine, Liverpool, UK; 2 Cochrane South Africa, South African Medical Research Council, Cape Town, South Africa; 3 Centre for Evidence-based Health Care, Faculty of Medicine and Health Sciences, Stellenbosch University, Cape Town, South Africa

**Keywords:** HIV/AIDS, Home-based care, Nurse-led care, Adherence to antiretroviral drugs, Psychosocial support

## Abstract

**Background:**

Home-based care is used in many countries to increase quality of life and limit hospital stay, particularly where public health services are overburdened. Home-based care objectives for HIV/AIDS can include medical care, delivery of antiretroviral treatment and psychosocial support. This review assesses the effects of home-based nursing on morbidity in people infected with HIV/AIDS.

**Methods:**

The trials studied are in HIV positive adults and children, regardless of sex or setting and all randomised controlled. Home-based care provided by qualified nurses was compared with hospital or health-facility based treatment. The following electronic databases were searched from January 1980 to March 2015: AIDSearch, CINAHL, Cochrane Register of Controlled Trials, EMBASE, MEDLINE and PsycINFO/LIT, with an updated search in November 2016. Two authors independently screened titles and abstracts from the electronic search based on the study design, interventions and types of participant. For all selected abstracts, full text articles were obtained. The final study selection was determined with use of an eligibility form. Data extraction was performed independently from assessment of risk of bias. The results were analysed by narrative synthesis, in order to be able to obtain relevant effect measures plus 95% confidence intervals.

**Results:**

Seven studies met the inclusion criteria. The trial size varied from 37 to 238 participants. Only one trial was conducted in children. Five studies were conducted in the USA and two in China. Four studies looked at home-based adherence support and the rest at providing home-based psychosocial support. Reported adherence to antiretroviral drugs improved with nurse-led home-based care but did not affect viral load. Psychiatric nurse support in those with existing mental health conditions improved mental health and depressive symptoms. Home-based psychological support impacted on HIV stigma, worry and physical functioning and in certain cases depressive symptoms.

**Conclusions:**

Nurse-led home-based interventions could help adherence to antiretroviral therapy and improve mental health. Further larger scale studies are needed, looking in more detail at improving medical care for HIV, especially related to screening and management of opportunistic infections and co-morbidities.

## Background

HIV/AIDS is a significant cause of morbidity and mortality in low and middle-income countries (LMICs), where health services already contend with poor infrastructure and limited resources including staff, drugs and equipment. Approximately 36.7 million people are living with HIV and around 52% of these are in Sub-Saharan Africa [1]. In 2015, an estimated 2.1 million people became newly infected with HIV/AIDS while 1.1 million of those with HIV/AIDS died [1].

The 2016 World Health Organisation (WHO) guidelines broaden the number of people eligible to start life-saving antiretroviral therapy (ART) [2]. Although these changes can improve clinical outcomes and reduce the incidence of HIV, they pose a challenge for public health services already overburdened with limited human and financial resources. Global coverage of antiretroviral therapy increased to 46% at the end of 2015 but disparities remain between high and low income countries [1].

Barriers to accessing and retaining care include amongst others transport costs, long waiting times and a shortage of healthcare staff [3]. Decentralising HIV treatment to community or home based settings and task-shifting using non-physician health workers for the initiation and maintenance of ART may help to overcome some of these problems, and these measures are being adopted as key management strategies [4–6].

### Home-based care

The WHO defines home-based care (HBC) as any form of care given to ill people in their homes, including physical, psychosocial, palliative and spiritual activities [7]. There are various types of HBC including integrated HBC where all service providers are involved, single service HBC involving one organisation, and informal HBC with no formal support structure [8]. HBC can be carried out by a variety of people including qualified healthcare practitioners, nurses, trained lay community health workers, peer health workers and HBC volunteers [9].

Providing care in the home can overcome some of the barriers to care, such as transport costs and waiting times, and help to reduce the burden on health facilities [10]. Other benefits of HBC include lower costs at both individual and country level, personalised care and being in familiar surroundings. It can also reduce demand on hospital beds and increase effective time use in hospitals [10].

### Home-based care for HIV

**Fig. 1:**
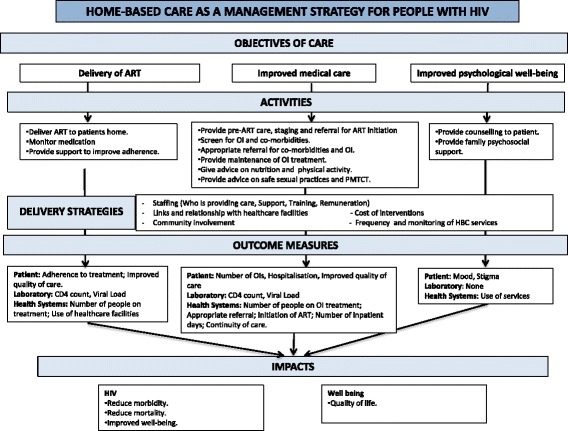
Home-based care as a management strategy for people with HIV

HBC has been defined by the Committee on a National Strategy for AIDS (CNSA) for the USA as care at the patient’s residence to supplement or replace hospital care including medical management, palliative care and social support [8]. HBC objectives for HIV/AIDS can include improved medical care, delivery of ART and improved psychosocial well-being (Fig. 1 Home-based care as a management strategy for people with HIV). It could have positive social outcomes by helping to reduce the stigma surrounding HIV, thereby improving support, access and adherence to ART and uptake of testing [10, 11]. An integrated approach using HBC to provide co-ordinated care for a number of conditions has been suggested, for instance combining HIV and tuberculosis management [12, 13].

Recent expansion of ART programmes has led to a growing emphasis on the decentralisation of HIV treatment in LMICs. Kredo assessed the effects of decentralised HIV care in relation to both initiation and maintenance of ART [5]. Wringe reviewed whether the conditions are in place to effectively scale up HBC programmes for increased ART, in terms of available human resources, health systems and funding mechanisms. They concluded that sustainable funding needs to be ensured, and policies to encourage staff retention and service integration are needed [10].

### Home-based care for other conditions

A number of reviews have examined the effects of home care for a variety of conditions. Okwundu found that home or community-based programmes for treating malaria could increase the number of people who receive appropriate anti-malarial treatment and may reduce all-cause mortality [14]. For cardiovascular disease, Clark found that home-based secondary prevention programmes conducted by health professionals are as effective as hospital based cardiac rehabilitation, and considerably lower in cost [15]. A study looking at end of life care at home for terminal patients supported the use of home care programmes for increasing the numbers of patients who will die at home [16]. In contrast, Smeenk concluded that the effectiveness of home care programmes for patients with terminal cancer remains unclear [17].

### Task-shifting

Task shifting from medical doctors to nurses is a potentially effective strategy to overcome the medical workforce shortage and to address the needs associated with chronic HIV/AIDS [13]. The increasing use of lay health workers to fill gaps in the work force raises the issues of training required and the quality of care provided. A review examining the use of lay health workers in the management of infectious diseases found them to be effective for improving tuberculosis outcomes but there was insufficient evidence to make a conclusion regarding adherence support in HIV/AIDS [18].

Kredo found that there is probably no reduction in the quality of care when trained nurses or community health workers initiate and maintain ART. When nurses initiate and maintain ART, there may also be lower loss to follow-up compared with doctors [6]. More research is needed into the level of training required and the ability of these workers to perform effectively whilst taking on multiple roles [9, 19].

### Why is it important to do this review?

In addition to the delivery of ART, other current key issues in the multidimensional care of HIV/AIDS include counselling and home-based testing, pre-ART care (such as repeating eligibility assessment), delivery of preventative interventions, distribution of prophylaxis, treatment of opportunistic infections and supportive psychosocial activities.

Young found a range of HBC models and interventions for HIV but these were generally from small studies and the majority were based in developed countries [20]. Since this review was published, there has been a change in the evidence with more focus on HBC in LMICs. Home-based HIV voluntary counselling and testing has been found to have the potential to increase uptake in developing countries [21]. Nurses are an important element of HBC for HIV but have not been covered in depth in Young or any other recent review. This review assesses the effects of home-based nursing to reduce morbidity in people infected with HIV/AIDS.

## Methods

### Criteria for considering studies for this review

We included all randomised controlled trials (RCTs) conducted amongst HIV/AIDS positive individuals, adults and children, comparing home-based care, including all forms of treatment, care and support offered in the HIV/AIDS positive person’s home by qualified nurses who have received a formal professional certificate or tertiary education degree, compared to hospital or health-facility based treatment. Home-based HIV voluntary counselling and testing was excluded as it is covered in Bateganya [21]. Initiation and delivery of ART was excluded as it is covered in Kredo [5]. We considered various outcomes including progression to AIDS, death, psychosocial outcomes (mood scores, stigma, patient and carer preferences), quality of care, quality of life, inpatient days, and number and type of opportunistic infections.

### Search methods for identification of studies

The following electronic databases were searched from 1980 to March 2015: Cochrane Register of Controlled Trials, MEDLINE, EMBASE, AIDSearch, CINAHL, PsycINFO/LIT, with an updated search in MEDLINE in November 2016. Detailed search strategies were compiled for each database searched (Table 1 details the MEDLINE search strategy). Clinicaltrials.gov and the WHO International Clinical Trials Registry Platform were searched in March 2015 to identify on-going trials. The strategy was iterative, in that references of included studies were searched for additional references. All languages were included.

**Table 1:** MEDLINE search strategy

Search	Most recent queries
#1	Search HIV Infections[MeSH] OR HIV[MeSH] OR hiv[tw] OR hiv-1*[tw] OR hiv-2*[tw] OR hiv1[tw] OR hiv2[tw] OR hiv infect*[tw] OR human immunodeficiency virus[tw] OR human immunedeficiency virus[tw] OR human 19rospe-deficiency virus[tw] OR human immune-deficiency virus[tw] OR ((human 19rospe*) AND (deficiency virus[tw])) OR acquired immunodeficiency syndrome[tw] OR acquired immunedeficiency syndrome[tw] OR acquired 19rospe-deficiency syndrome[tw] OR acquired immune-deficiency syndrome[tw] OR ((acquired 19rospe*) AND (deficiency syndrome[tw])) OR “Sexually Transmitted Diseases, Viral”[MeSH:NoExp]
#2	Search randomized controlled trial [pt] OR controlled clinical trial [pt] OR randomized controlled trials [mh] OR random allocation [mh] OR double-blind method [mh] OR single-blind method [mh] OR clinical trial [pt] OR clinical trials [mh] OR (“clinical trial” [tw]) OR ((singl* [tw] OR doubl* [tw] OR trebl* [tw] OR tripl* [tw]) AND (mask* [tw] OR blind* [tw])) OR (placebos [mh] OR placebo* [tw] OR random* [tw] OR research design [mh:noexp] OR comparative study [mh] OR evaluation studies [mh] OR follow-up studies [mh] OR prospective studies [mh] OR control* [tw] OR 19rospective* [tw] OR volunteer* [tw]) NOT (animals [mh] NOT human [mh])
#3	Search Home-based care or homebased care or home based care
#4	Search Home care or homecare or home-care
#5	Search Home
#6	Search #3 OR #4 OR #5
#7	Search #1 AND #2 AND #6

### Selection of studies, data collection and analysis

Titles, abstracts and descriptor terms of the electronic search results were screened independently by two authors for relevance based on predefined eligibility criteria. Full text articles were obtained of all selected abstracts and final eligibility assessed. Data including administrative details, study design, details of the intervention and control, and outcomes were extracted independently by two authors using a standardised data extraction form.

The risk of bias of included studies was also evaluated independently by two authors using the Cochrane risk of bias tool. We assessed and summarised the following main items in the ‘Risk of Bias’ table: sequence generation, allocation concealment, blinding of participant and personnel, blinding of outcome assessment, whether incomplete outcome data were adequately addressed, selective reporting and any other bias.

Missing or inadequate data were addressed by contacting authors. We resolved disagreements by discussion.

Relevant effect measures and the 95% confidence intervals (CI) were reported. We planned to assess sources of clinical and methodological heterogeneity by looking at characteristics of studies, evaluating similarity between type of participants, intervention used and outcomes. However, due to the varied outcomes used in the included studies no meta-analysis could be conducted.

## Results

### Description of studies

**Fig. 2:**
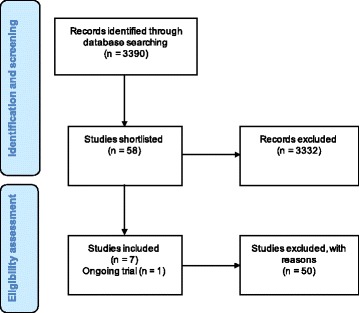
Flow diagram of study selection

Seven studies met the inclusion criteria (Fig. 2 Flow diagram of study selection). In addition, we identified one on-going trial currently being conducted in Kenya investigating home-based directly observed ART [22]. Fifty studies were excluded. Reasons for exclusion are detailed in Table 2.

**Table 2:** Table of excluded studies

Reason for exclusion	Number of studies [references]
Not all participants are HIV positive	3 studies [34–36]
Intervention studied was not home-based care	5 studies [37–41]
Intervention was not provided by qualified nurses	15 studies [42–56]
Comparison of two models of home-based care	2 studies [57, 58]
Study investigating home-based voluntary HIV counselling and testing	12 studies [59–70]
Not a randomised controlled trial	12 studies [71–82]
Trial terminated for non-compliance with human subject regulations	1 study [83]

Individuals were randomised in each of these seven studies. Trial sizes varied from *n* = 37 to *n* = 238. Only one trial was conducted in children [23] whilst the rest were conducted in adults (≥18 years). One trial focused on female adults [24] and the others included males and females. Five studies were conducted in the USA [23–27] and two in China [28, 29].

The interventions studied and outcomes measured varied between studies (Table 3). Four studies could be classified under home-based adherence support [23, 27–29]. Five studies investigated providing home-based psychosocial support [24–26, 28, 29].

**Table 3:** Description of included studies

Category	Type of intervention and comparison	Study ID	Participants	Follow-up duration	Study location	Outcomes
Adherence support	Home-based nursing vs. standard care	Berrien 2004 [23]	HIV-positive children. *N* = 37.	12 months.	Connecticut, USA.	Adherence; Viral load; CD4 count.
		Wang 2010 [28]	HIV-positive adults; active or previous heroin addicts; on ART at least 1 month prior to starting study. *N* = 116.	10 months.	Hunan, China.	Adherence.
		Williams 2006 [27]	HIV-positive adults on ART. *N* = 171.	15 months.	Connecticut, USA.	Adherence; Viral load; CD4 count.
		Williams 2014 [29]	HIV-positive adults; on ART; self-reported < 90% adherence to pre-ART medications or to ART; willing to receive home visits. *N* = 110.	12 months.	Hunan, China.	Adherence; Viral load; CD4 count.
Improved psycho-social wellbeing	Home based nursing vs. standard care	Blank 2014 [25]	HIV-positive adults; understand spoken English; had a diagnosed serious mental illness; able to provide informed consent. *N* = 238.	24 months.	Philadelphia, USA.	Health related quality of life; Viral load; CD4 count.
		Hanrahan 2011 [26]	HIV-positive adults; lived within the city limits of Philadelphia; had a diagnosed serious mental illness (SMI). *N* = 238.	12 months.	Philadelphia, USA.	Psychiatric symptoms; Health related quality of life
		Miles 2003 [24]	African American women with HIV who were primary caregivers for at least one child under the age of 9 years. *N* = 109.	6 months.	USA.	Emotional distress (depression, stigma, worry); Health related quality of life
		Wang 2010 [28]	HIV-positive adults; active or previous heroin addicts; on ART at least 1 month prior to starting study. *N* = 116.	10 months.	Hunan, China.	Quality of life, Depression
		Williams 2014 [29]	HIV-positive adults; on ART; self-reported < 90% adherence to pre-ART medications or to ART; willing to receive home visits. *N* = 110.	12 months.	Hunan, China.	Depressive symptoms; social support; HIV stigma scale.

Detailed information on the participants, interventions, controls and outcomes can be found in Table 3.

### Risk of bias in included studies

The risk of bias assessment is summarised in Table 4.

**Table 4:** Risk of bias of included studies

Bias	Berrien 2004 [23]	Blank 2014 [25]	Hanrahan 2011 [26]	Miles 2003 [24]	Wang 2010 [28]	Williams 2006 [27]	Wiliams 2014 [29]
Random sequence generation	Small table of random digits.	Randomised on a 1:1 basis but method unclear.	Computer-generated algorithm	Table of random numbers.	Not reported.	Stratified randomisation, with block size of 10.	Stratified randomisation, with block size of 10.
Allocation concealment	Randomisation list held by clinical coordinator of HIV program, kept in a locked file.	Not reported.	Person allocating different from the one assessing the inclusion.	Not reported.	Not reported.	Not reported.	Not reported.
Incomplete outcome data	Lost to follow-up: 5% intervention; 11% control.	Lost to follow up not reported.	Lost to follow-up: 10% intervention; 5% control.	Lost to follow-up at 6 months: 51% intervention; 58% control.	Lost to follow-up: 14% intervention; 17% control.	Lost to follow-up at 12 months: 28% intervention; 25% control.	Lost to follow-up at 12 months: 5% intervention; 22% control.
Selective reporting	All outcomes reported.	All outcomes reported.	All outcomes reported.	All outcomes reported.	All outcomes reported.	All outcomes reported.	Incomplete reporting of social support and stigma.
Blinding of participants and personnel	None.	Participants not blinded.	None.	None.	Not reported.	All personnel were blinded except the home visit team. Participants not blinded.	None.
Blinding of outcome assessment	None.	Research staff blinded. Participants disclosed information, unmasking experimental status.	Data collectors blinded.	Data collectors blinded.	Not reported.	All personnel including interviewers were blinded except the home visit team.	Not reported.

#### Allocation (selection bias)

All trials stated that participants were randomised. Five trials used adequate methods for generating the allocation sequence. Of these five trials, two used random number tables [23, 24], one used a computer generated algorithm [26] and two used a stratified randomisation procedure [27, 29]. Allocation concealment was adequate in two trials [23, 26] and not reported in the remaining trials.

#### Blinding (performance bias and detection bias)

In Williams 2006 all personnel (except the home intervention team) and the interviewers were blind throughout the course of the study whereas in Hanrahan and Miles, only the data collectors were blinded [24, 26, 27]. Blank was designed to be single blind for the research staff but participants sometimes disclosed information, which made it possible to identify their experimental status [25].

In Williams 2014 there was no blinding of the participants but it was unclear whether the outcome assessors were blinded [29]. Berrien used no blinding and Wang did not report blinding [23, 28].

#### Incomplete outcome data (attrition bias)

Three trials reported a loss to follow up of less than 20% and two reported a loss to follow up of less than 30% [23, 26, 28]. Miles had a high loss to follow up of 51% in the intervention group and 58% in the control group [24]. One study did not report loss to follow up [25].

#### Selective reporting (reporting bias)

All outcomes stated were reported.

### Effects of interventions

The effects of the various interventions are summarised in Table 5.

**Table 5:** Summary of results

	Outcomes	Summary
Adherence support	Adherence	-Self-reported adherence improved with intervention [23, 28, 29]. -Intervention increased ratio of the number of recorded Medication Event Monitoring Systems (MEMS) cap openings to the number of openings to be expected if the medication were taken as prescribed [27]. -Pharmacy drug refill increased significantly with intervention [23].
	Viral load	No significant change [23, 27, 29].
	CD4 count	No significant change [23, 27, 29].
Improved psycho-social wellbeing	Health-related quality of life	-SF-12 mental health subscale improved with intervention but not the SF-12 physical health subscale [25]. -No clear difference in health related quality of life outcomes (SF-12) [26]. -Improved WHO quality of life measures [28].
	Psychiatric symptoms	No significant difference between groups in reduction in psychiatric symptoms (Colorado Symptom Index (CSI) score) [26].
	Emotional distress (depression, stigma, worry)	-Reduced symptoms of depression (PHQ-9 score) with intervention [26]. -Reduced HIV stigma, worry, physical functioning but no significant difference in depressive symptoms, mood, general health or overall functioning [24]. -Reduced symptoms of depression (Self-rating Depression Scale) [28]. -Reduced symptoms of depression (CESD scale) in intervention group. No significant difference in social support (SSRS) and stigma (HIV stigma scale) [29].

#### Adherence support

Four studies, one in children [23] and three in adults [27–29] examined the effects of an intensive home based nursing programme compared to standard care on adherence to ART. Berrien, Williams 2006 and Williams 2014 also reported viral load and CD4 counts [23, 27, 29]. However, due to differences in the way these three outcomes were measured and reported we could not do a meta-analysis.

In Berrien’s study (*n* = 37) a home care registered nurse made eight pre-planned home visits over three months, aiming to increase patient understanding of HIV infection and ARVs and prevent issues with adherence [23]. Standard care in a clinic included the doctor, nurse, and social worker providing customary medication adherence education. Medication adherence measured by pharmacy drug refill was significantly better (mean refill score 2.7 in the intervention group and 1.7 in control group, *p* = 0.002). The intervention group also showed improvement in their knowledge score compared to the control group (*p* = 0.02) and in their reported adherence although this difference was not statistically significant (*p* = 0.07).

There were no significant differences in the change in CD4 counts or viral loads either immediately or 6–11 months later. 45% of participants in the intervention group maintained or achieved a viral load, < 2.6 log_10_copies/ml (<400copies/ml) compared to 24% in the control group. CD4 figures were not reported [23]. This study had a low risk of selection and attrition bias but the small sample size of the study reduces the quality of these results.

Wang (*n* = 116) provided nurse-delivered home visits combined with telephone calls to HIV-infected heroin users to the intervention group, whilst the control group received routine care involving a monthly clinic visit [28]. Assessment of selection, performance and detection bias was not possible due to lack of reporting of these methodological aspects. There was a low risk of attrition bias and at the end of the eight months, participants in the experimental group were more likely to report taking 100% of pills (Mantel-Haensel 1.57, 95% CI 1.19 to 2.07, reported *p* = 0.0001) and taking pills on time (Mantel-Haensel 2.50, 95% CI 1.51 to 4.13, reported *p* = 0.0001) than those in the control group.

Williams 2006 (*n* = 171) assessed an adherence tool which followed a structured educational model facilitated by a nurse and community support worker conducting home visits, compared to standard care [27]. Adherence was recorded as the ratio of the number of Medication Event Monitoring Systems cap (MEMS cap) openings to number of openings expected if the medication was taken as prescribed. There was a low risk of detection bias, loss to follow-up of 28% and 25% respectively, and allocation concealment was not reported. The median CD4 counts were 345 and 341 for the intervention and control arms respectively. Comparing the proportion of participants with greater than 90% adherence, there was a statistically significant difference between the two arms at 15 months, favouring the intervention group (reported extended Mantel-Hansel test 5.80, *p* = 0.02). There were no significant changes at 12 and 15 months in proportion of people with CD4 count greater than 200 or undetectable viral loads (actual figures not reported).

Williams 2014 (*n* = 110) investigated the effects of an adherence intervention, which included home-based social and educational components provided by a nurse and peer educator, compared to standard care adherence clinic support services [29]. A visual analogue scale was used to evaluate adherence to ART over the previous 30 days. There is a risk of attrition bias with differential loss to follow-up of 5% and 22% at 12 months. At baseline all subjects reported taking 90% or less of prescribed medication (pre-ART or ART). In reported bivariate analyses, there was a significant difference in adherence between the two groups at 6 and 12 months (*p* = 0.003 and *p* = 0.005). In reported multivariate analyses, controlling for baseline factors, the experimental group had a significantly higher proportion of people who were adherent (*p* = 0.009). The proportion of those with an undetectable viral load increased in both groups at 6 months (44% in control group, 57% in intervention group) and at 12 months (59% control group, 72% intervention group). But in multivariate analyses controlling for baseline factors, there was no difference between groups (*p* = 0.18). In reported multivariate analyses CD4 count did not differ by group (*p* = 0.65) but an overall increase in CD4 count category was seen in all subjects between baseline and 12 months (*p* = 0.003).

#### Psychosocial support

Five studies investigated the effects of a home-based programme provided by nurses on psychological wellbeing [24–26, 28, 29].

### Psychiatric care

Blank (*n* = 238) examined a home-based advanced practice psychiatric nurse intervention in individuals with serious mental illness and HIV [25]. The nurse provided in-home consultations and co-ordinated medical and mental health services. Growth curve analysis estimating the treatment effects on viral load (log_10_copies/ml) and Medical Outcomes Study 12-Item Short-Form Health Survey (SF-12) mental health scores, showed that the intervention reduced the rate of change over time in viral load and increased the rate of change over time in SF-12 mental health outcomes. They also compared CD4 percentage and the SF-12 mental health score, showing only significant treatment effect for SF-12 mental health score. A model comparing viral load and SF-12 physical health scores showed a significant decline in viral load but no change in perceptions of physical health status. A model comparing CD4 and SF12 physical health showed no significant change. Therefore, results show significant improvement in health-related quality of life for a mental health subscale but not for a physical health subscale. However, there was a risk of both detection and performance bias.

Hanrahan (*n* = 238) evaluated a home-based advanced practice psychiatric nurse intervention in individuals with serious mental illness and HIV [26]. The nurse provided in-home consultations and co-ordinated medical and mental health services. Both groups had a reduction in psychiatric symptoms (Colorado Symptom Index (CSI) score) from baseline to 12 months, but the relative difference in these improvements was not significant (reported effect (d) = − 4.03, 95% CI -15.99 to 7.83, *p* = 0.51). However, during the same period symptoms of depression (PHQ-9 score) significantly decreased in the experimental group compared to the control group, with an average treatment effect of an increased PHQ-9 score of 4.40 (95% CI -2.66 to 11.46, *p* = 0.2). There was no clear difference in changes in health-related quality of life outcomes (SF-12) over time between groups. In repeated measured random regression models, Group x Time interactions were all non-significant (reported *p* > 0.05). Allocation concealment was adequate and data collectors were blinded.

### Counselling and emotional support

Miles (*n* = 109) assessed the psychosocial impact of home visits carried out by three registered nurses in the homes of African American women with HIV who were the principal caregivers for one or more children under the age of nine [24]. At six months, there was a statistically significant difference in scores for physical functioning (Medical Outcomes Survey-HIV (MOS-HIV)) (WMD 1.45, 95% CI 0.01 to 2.89), HIV stigma (Demo HIV stigma scale) (WMD -0.25, 95% CI -0.49 to − 0.01) and HIV worry (HIV worry scale) (WMD -0.46, 95% CI -0.89 to − 0.03). For depressive symptoms (Centre for Epidemiological Studies Depression (CESD) scale), mood (Profile of Mood States (POMS)), general health, and overall functioning (MOS-HIV), no statistically significant difference was found. This study however had a high loss to follow up which weakens the quality of results.

Wang (*n* = 116) examined the effects of nurse-delivered home visits combined with telephone calls on the quality of life of HIV-infected heroin users [28]. The intervention had a significant effect in reducing the symptoms of depression (assessed using Chinese version of Self-rating Depression Scale (SDS)) (MD -11.53, 95% CI -17.74 to − 5.92). Quality of life measures (measured by the Chinese version of WHO Quality of Life) also improved including physical (MD 2.97, 95% CI 1.75 to 4.19), psychological (MD 2.72, 95% CI 1.61 to 3.83), social (MD 2.10, 95% CI 0.78 to 3.42) and environmental (MD 2.40, 95% CI 1.23 to 3.57) domains.

Williams 2014 (*n* = 110) investigated the effects of nurse-delivered home visits on symptoms of depression (CESD scale), social support (Social Support Rating Scale (SSRS)) and stigma (HIV stigma scale) [29]. At baseline, reported multivariate analyses adjusting for baseline social support, stigma and raw CESD score, showed a significant difference in overall depression scores between the two groups (*p* = 0.001), with the control group having a higher percentage of people with CESD score ≥ 16. When baseline CESD scores were compared to 12-month CESD scores, they reported a significant decrease in depressive symptoms in the intervention group compared to the control group (*p* = 0.03). There was also a significant change between the six-month CESD scores and 12-month CESD scores, with an increase in depressive symptoms in control subjects and decrease in intervention subjects (*p* = 0.05). Baseline raw CESD and baseline stigma were significant predictors of CESD scores (*p* < 0.001 and *p* = 0.003 respectively) but not baseline social support.

## Discussion

Over the past five years various reviews have assessed community-based care for HIV (Table 6). Interventions have looked at decentralised treatment and use of lay health workers and volunteers. The WHO recommends that nurse-led teams can deliver most interventions including initiating and monitoring ART, managing uncomplicated opportunistic infections and providing primary mental health and neurological care [30]. Our review adds to what is known by focussing on assessing the effects of home-based nursing on morbidity in people with HIV/AIDS.

**Table 6:** Summary of systematic reviews on community-based care for HIV

Review	Date of search, number of included studies	Participants	Intervention	Comparison	Outcomes	Summary of key findings
Decroo 2013 [84]	Feb 2013. 18 studies: 2 cluster RCTs, 11 prospective/ retrospective cohort studies, 2 qualitative studies, 1 cost-effectiveness study, 1 activity report from an NGO, 1 abstract.	PLWHA	-Home-based ART delivery by CHWs. -Home-based ART delivery by volunteers. -Home-based ART by peer CHWs. -Patient-led community ART dispensing.	Facility based ART	-Attrition on ART. -Virological rebound on ART. -Cost –health service costs, patient costs. -Social.	-Increase adherence and accessibility to AR. -Cost effective -Positive social outcomes
Kredo 2013 [5]	March 2013. 16 studies: 2 RCTs, 14 cohort.	HIV-infected patients at point of initiating treatment and patients already on treatment requiring maintenance and follow-up.	Any form of decentralised care delivery model for initiation or continuation of treatment, or both.	Care delivered at centralised site (usually a hospital or health facility)	-Attrition (composite of loss to follow up or death). -Loss to follow up at set time points after intervention. -Death. -Time to starting ART. -Patients diagnosed with TB after entry into HIV care. -Virologic response to ART (viral load). -Immunological response to ART (CD4+). -Occurrence of new AIDS-defining illness. -Patient satisfaction with care. -Cost to provider. -Cost to patient and family. -Any negative impact on other programme and health care delivery.	-Lower attrition in partial decentralisation models (ART started in hospital and continued at health centre). -No difference in attrition in full decentralisation models (ART started and continued at peripheral health centre) but fewer patients lost to care. -No difference in outcomes detected for ART provided at home by trained volunteers compared to facility-based care.
Mwai 2013 [85]	December 2012. 21 studies: 5 qualitative, 7 cohort, 6 mixed method, 3 RCTs.	PLWH	CHWs in HIV	Facility based HIV care	Patient related: -Knowledge and literacy of HIV -Behaviour change -Uptake of HIV and other services. -Adherence to ART. -Retention in care. -Viral suppression. -Mortality. -Socio-economic status and quality of life. -Palliative care. Health system: -Service organisation and delivery -Data collection, surveillance and reporting -Service cost	CHWs perform a variety of roles in HIV including counselling, testing, home-based care, education, adherence support, livelihood support, screening, referral and surveillance activities, retention in care. No evidence that patient outcomes and quality of care are compromised. CHWs may also have positive impacts on HIV service organisation, delivery and cost. But to be sustainable, need to be better integrated into wider health systems.
Nachega 2016 [86]	January 2016. 22 studies: 11 RCTs, 11 cohort.	HIV-infected individuals initiated on ART.	Community-based ART delivery.	Health-care facility (e.g. hospital or clinic)	-Proportion of PLWHA with optimal ART adherence levels (> 80%). -Proportion of PLWH with virologic suppression at 12 and/or 24 months after ART initiation. -Engagement (proportion of patients retained in care at 12 and/or 24 months post-ART initiation). -All-cause mortality. -Reported stigma. -Cost to patient and provider and cost effectiveness.	-No significant difference in optimal ART adherence, virological suppression, all-cause mortality and loss to follow-up between 2 groups when analysis was restricted to RCTs. -Pooled analysis from both RCTs and cohort studies showed higher rates of retention in care in community-based ART group than facility-based group. -Only 2 eligible studies reported on cost or cost-effectiveness outcomes. These suggest that community-based ART services may be more cost-effective in the long run but more research using economic outcomes is needed.
Rachlis 2013 [87]	December 2011, updated February 2012. 21 CBC programs	Urban /rural populations including PLWHA, their family members, orphans, vulnerable children.	Community-based care (CBC) programmes		-Region -Vision -Characteristics of target population -Program scope (services provided) -Program operations -Funding models -Human resources -Sustainability -Monitoring and evaluation.	9 key categories useful for describing and organising CBC HIV/AIDS programs in resource limited settings. Suggest can be used to inform potential logic models to enhance overall program performance and to develop evidence based tools for sustainable HIV/AIDS service delivery.
Wouters 2012 [9]	December 2011. 30 studies: 9 descriptive, 4 quasi-experiments, 5 retrospective/observational cohort studies, 2 qualitative, 6 (cluster/nested) RCTs.	PLWHA	9 types community support providers: -CHWs (non-professional healthworkers who undertake short course training, work in own communities to support services provided by other health workers) -Peer health workers (CHWs who are HIV positive). -Field officers -Health extension workers -HIV/AIDS lay counsellors -DOT for ART -Adherence supporters -HBC volunteers	Health facility based care	ART programme outcomes: -Access and increasing coverage of ART programmes -Adherence -Virological/ immunological -Patient retention -Survival rates Contributory role of community program: -Integration of ART services into general health system. -Providing psychosocial care. -Empowered ART patients towards self-management. -Defaulter tracing. -Community as a resource.	Community support can positively impact ART programme delivery and outcomes in resource-limited settings. Potential strategy to address shortage of health workers/ broaden care to accommodate needs associated with chronic HIV/AIDS. More research needed to understand which tasks performed by community support initiatives contribute to long-lasting ART success and limits to which lay health workers can assume multiple roles.

To minimise publication and indexing bias, we used two authors working independently to select studies, perform data extraction and risk of bias assessments. The review included seven studies, conducted in the USA and China. Included studies were small, conducted mainly in adults. Five trials used adequate methods for generating the allocation sequence [23, 24, 26, 27, 29] and allocation concealment was adequate in two trials [23, 26]. Only one trial reported high losses to follow up [24]. This type of intervention is difficult to blind and only one trial blinded personnel and data collectors [27]. Another limitation is that grey literature was not searched.

Reported adherence to ART improved with nurse-led home-based care. Self-reported adherence measures could be liable to a social desirability bias in which participants felt obliged to report that their adherence was better at the end of the trial because they were aware that this is what the intervention is aiming to achieve. However more objective measures to adherence such as the pharmacy drug refill did also support this. Despite improved adherence to ART, the interventions did not appear to affect biological parameters. Interestingly a review of direct observation in HIV therapy, mostly performed in the community, also reported no effect on virological suppression [31]. Possible reasons for a high viral load despite adherence include drug resistance, treatment failure or an adequate adherence irrespective of the intervention [31]. Another systematic review examining interventions for enhancing adherence to ART also concluded that nurse-led home-based strategies are effective [32].

In terms of psychological support, psychiatric nurse home interventions improved depressive symptoms in those with an existing mental health condition. Other findings for home based psychological support were mixed. Two studies found a reduction in symptoms of depression, whilst another found no difference, but stigma scales and physical functioning were improved. This highlights the potential psychosocial benefits of home-based care but with the self-reported scales, there could also be an element of the social desirability bias mentioned above, particularly as participants were not blinded. Wouters 2012 also found that community-based initiatives providing social support and counselling to people living with HIV could effectively support and improve medical care [9].

None of the included studies looked at improved medical care (Fig. 1). With the current move to test and treat, pre-ART care is now not so relevant but other medical care such as screening for opportunistic infections and co-morbidities and related referral is important. Oni et al. found a high prevalence of multiple morbidities among ART patients aged under 45 years [33]. With the increase in diseases of lifestyle, such as diabetes and hypertension, early identification and management of co-morbidities is also important [33].

## Conclusions

The results indicate that nurse led home-based interventions could help adherence to ART. Psychiatric nurse support in those with existing mental health conditions improved mental health and depressive symptoms. Home-based psychological support impacted on HIV stigma, worry and physical functioning and in certain cases depressive symptoms.

However, studies were generally small and conducted in limited geographic areas, limiting the quality of evidence. Further larger scale studies are needed looking in more detail at improving home-based medical care for HIV, for example screening for opportunistic infections and co-morbidities.

## Abbreviations

AIDS: acquired immunodeficiency syndrome
ART: antiretroviral therapy
CD4 cell: T helper cell (type of white blood cell) that is a vital part of the human immune system
CESD: Centre for Epidemiological Studies Depression Scale
CHW: community health worker
CI: confidence interval
CNSA: Committee on a National Strategy for AIDS
DOT: directly observed treatment
HBC: home-based care
HIV: human immunodeficiency virus
LMIC: low and middle income country
MEMS: Medication Event Monitoring Systems
MOS-HIV: Medical Outcomes Survey-HIV
PLWHA: People living with HIV/AIDS
RCT: randomised controlled trial
SF-12: Medical Outcomes Study 12-Item Short-Form Health Survey
WHO: World Health Organisation
WMD: weighted mean difference
